# Triacontanol Reduces Transplanting Shock in Machine-Transplanted Rice by Improving the Growth and Antioxidant Systems

**DOI:** 10.3389/fpls.2016.00872

**Published:** 2016-06-17

**Authors:** Xiaochun Li, Qiuyi Zhong, Yuxiang Li, Ganghua Li, Yanfeng Ding, Shaohua Wang, Zhenghui Liu, She Tang, Chengqiang Ding, Lin Chen

**Affiliations:** Jiangsu Collaborative Innovation Center for Modern Crop Production/National Engineering and Technology Center for Information/Agriculture/Key Laboratory of Crop Physiology and Ecology in Southern China, Nanjing Agricultural UniversityNanjing, China

**Keywords:** machine-transplanted rice, transplanting shock, recovery stage, tiller, triacontanol (TRIA), antioxidant systems

## Abstract

Machine transplantation results in serious transplant shock in seedlings and results in a longer recover stage, which negatively impacts the growth of low-position tillers and the yield of machine-transplanted rice. A barrel experiment was conducted to examine the effect of the foliar application of triacontanol (TRIA) on machine-transplanted rice during the recovery stage. TRIA (0, 1, 5, and 10 μM) was sprayed over leaves 2 days before transplanting. The chlorophyll content, sucrose content, oxidative damage, antioxidant enzyme levels, glutathione (GSH), and ascorbate (ASA) redox states, tiller dynamics and yield components of the plants were investigated. The results show that foliar-applied TRIA significantly alleviates the growth inhibition and oxidative damage caused by transplant shock. Furthermore, the application of TRIA increased the chlorophyll and sucrose contents of the plants. Importantly, TRIA not only significantly improved the activity of catalase (CAT) and guaiacol peroxidase (POD), demonstrating that POD can play an important role in scavenging H_2_O_2_ during the recovery stage, but it also enhanced the redox states of ASA and GSH by regulating the activities of enzymes involved in the ASA–GSH cycle, such as ascorbate peroxidase (APX) and glutathione reductase (GR). A dose of 10 μM TRIA was the most efficient in reducing the negative effects of transplant shock, increasing the panicles, grain filling, and grain yield per hill by 17.80, 5.86, and 16.49%, respectively. These results suggest that TRIA acts to reduce transplant shock in association with the regulation of the redox states of ASA and GSH and antioxidant enzymes and serves as an effective antioxidant to maintain photosynthetic capacity and promote the occurrence of low tillers.

## Introduction

Rice, the most important food crop worldwide, is a staple food crop for more than 50% of the world’s population (Khush, 2005). An increase in rice production of 1.2% per year is required to meet the growing demand for food due to population growth and economic development over the next decade (Fageria et al., 2003). Rice is also one of the major food crops in China, being a staple food for more than 65% of its population. Therefore, improving rice production plays an important role in the food security of China. With the transfer of the rural labor force and the improvement and promotion of agricultural machinery and agronomic practices, the importance of mechanized rice planting has become increasingly prominent (Valipour, 2014).

Transplanting has a considerable impact on rice growth. The transplanting procedure generally delays the development of rice. Seedling roots are injured by transplanting, and the seedlings develop an imbalance between water uptake and transpiration. As a result, seeding leaves wilt due to water stress, and under severe stress, some leaves may die (Yamamoto, 1989; Yamamoto and Hisano, 1990). Various metabolic processes in transplanted seedlings are also disturbed by the influence of the changes in water content and root pruning (Sasaki and Gotoh, 1999). Consequently, the growth and development of seedlings temporarily stagnates. This phenomenon is called transplant shock.

The period of time during which plants are gradually restored to their normal physiological condition and growth after being transplanted is known as the recovery stage. The extent and duration of transplant-shock injuries affects crop growth and yield. Because the roots of blanket-like seedlings are always cut and split by transplanting, the machine transplanting of rice is more harmful than hand transplanting rice. Shun et al. (2005) found that the recovery stage of machine-transplanted rice is 2–3 days longer than that of hand-transplanted rice, with no increase in biomass 10 days after transplanting. Yuan et al. (2007) indicated that machine-transplanted rice postpones tiller development compared to those of hand-transplanted rice. Furthermore, a significant difference in rice grain yield is observed as the timing and growth patterns of initiation differ among different tillers (Wang et al., 2007). Low-position tillers, compared to high-position tillers, exhibit a higher production capacity (Zhao, 1993). Zhang et al. (2010) also indicated that the spikelet number per panicle, the grain weight, and the yield per spike decrease as the tiller position shifts from low to high. Therefore, relieving the transplant shock of machine-transplanted rice, shortening the recovery stage and promoting the growth of low tillers are all important to improving the yield of machine-transplanted rice.

Triacontanol (TRIA), a long-chain primary alcohol (C_30_H_61_OH), is a potent plant growth-promoting substance that is used for a number of agricultural and horticultural crops. TRIA improves plant growth, as well as the yield and quality characteristics of various crops (Ries, 1985), and it increases the rate of several biochemical and physiological processes (Ries and Houtz, 1983; Ries, 1991; Naeem et al., 2009, 2010). For example, triacontanol (TRIA) increases the contents of total chlorophyll (Chl), Chl a and Chl b by 25.1, 26.1, and 22.4%, respectively, after 4 h compared with the contents of control rice seedlings (Chen et al., 2003). The most profound effects of TRIA are that it increases growth, biomass, and photosynthetic activity, as well as the free amino acid, reducing sugar, and soluble protein content (Muthuchelian et al., 1995). Furthermore, TRIA contributes to the amelioration of negative impacts caused by abiotic stress. TRIA efficiently reduces the negative effects of salinity stress, and improves the photosynthetic rate, the transpiration rate, and the chlorophyll contents (Perveen et al., 2013).

Considering all of the major roles of TRIA in plants and the negative impact of transplant shock on seedlings, it was hypothesized that the foliar application of TRIA before mechanical-transplanting could reduce the negative effect of transplant shock. This study was performed to investigate the role of TRIA and its effects on the yield of machine-transplanted rice.

## Materials and Methods

### Plant Materials and Experimental Design

The experiments were carried out in the Agricultural Experiment Laboratory of Nanjing Agricultural University, Danyang, Jiangsu Province, China (32°00′N, 119°32′E, 7 m altitude) during the rice growing season from late May to late October in 2015. The local conventional japonica rice Wuyunjing 24 was sown in a seedling bed (28 cm × 58 cm) on May 28, 2015. The seeding rate was 100 g of seeds per seedling bed, and seedlings were transplanted into plastic barrels on June 20, 2015. The height and inner diameter of the plastic barrels were 35 cm and 34 cm, respectively. Each barrel contained 15 kg of soil up to a height of approximately 25 cm.

Two days before transplanting, the experimental treatment was conducted using different concentrations of triacontanol (TRIA): 0, 1, 5, and 10 μM. The seedling bed was sprayed with 200 ml of the treatment solution, and each treatment was repeated three times. The TRIA solutions were prepared in hot, distilled water with 0.1% Tween-20 solution. At transplanting, seedlings at similar growth stages were selected for simulated machine transplanting by pruning their roots down to 2 cm and transplanting them into plastic barrels. Each plastic barrel had 4 hills, with 4 seedlings per hill. The phosphorus, potassium and nitrogen contents were the same in each treatment, and the N: P_2_O_5_: K_2_O ratio was 1:0.5:0.8. The N rate was 1.8 g per barrel in each treatment, for which the ratio of three applications of urea was 3:3:4, including the basal fertilizer, tiller fertilizer, and spike fertilizer. Calcium super phosphate served as the P fertilizer, and K chloride served as the K fertilizer. The experiments were performed outdoors, and other management practices agreed with the local protocols for high-yield management.

### Parameter Measurements

#### Plant Growth

At 4, 8, and 12 days after transplanting, 10 plants were selected from each repeat, and all of the materials were rinsed three times in distilled water. The number of roots and green leaves, as well as the stem diameter, were measured. The plants were oven-dried at 105°C for 30 min, followed by 80°C for 72 h to a constant weight. Finally, fully expanded leaves were immediately snap-frozen in liquid nitrogen and stored at -40°C until analysis.

#### ROS Production and Membrane Damage

The leaf samples (0.1 g) were homogenized with a mortar and pestle in 5 ml of ice-cold phosphate buffer (50 mM, pH 7.8) containing 1% (w/v) insoluble polyvinylpolypyrrolidone (PVPP) (Jiang and Zhang, 2001). The extract was centrifuged at 16,000 *g* for 20 min at 4°C. The supernatant analyzed for H_2_O_2_ and MDA content. The content of H_2_O_2_ was estimated using a kit provided by the Nanjing Jiancehng Biology Company. Oxidative damage to membrane lipids was determined by measuring the level of MDA using a thiobarbituric acid (TBA) test as described by Jiang and Zhang (2001). The supernatant (1 ml) was homogenized in 5 ml of 10% (w/v) trichloroacetic acid (TCA) and centrifuged at 5000 *g* for 10 min, and the absorbance of the supernatant was monitored at 440, 532, and 600 nm. The levels of MDA were calculated using a molar extinction coefficient of 0.155 mM cm^-1^

#### Chlorophyll and Sucrose Contents

The method described by Arnon (1949) was used to determine the Chl a and b contents. Fresh leaves (0.5 g each) were cut into small pieces, to which 10 ml of 80% acetone was added, and samples were kept overnight at 0–4°C. The extract was centrifuged at 10,000 *g* for 5 min, and the absorbance (OD) of the supernatant was read at 645 and 663 nm with a UV-visible spectrophotometer.

Chl a and Chl b contents were calculated using the following formulae:

$$\begin{array}{c} \mathrm{Chla}=[12.7(OD663)-2.69(OD645)]\times v/1000\times w\\ \mathrm{Chlb}=[22.9(OD645)-4.68(OD663)]\times v/1000\times w \end{array}$$

v – volume of the extract [mL];

w – mass of the fresh leaf tissue [g].

The sucrose content was determined according to Zhang and Qu (2003) and was recorded as the decrease in absorbance at 485 nm.

#### Antioxidant Enzyme Activity

The leave samples (0.1 g) were homogenized with a mortar and pestle in 5 ml of ice-cold phosphate buffer (50 mM, pH 7.8) containing 1% (w/v) insoluble polyvinylpolypyrrolidone (PVPP) (Jiang and Zhang, 2001). The extract was centrifuged at 16,000 *g* for 20 min at 4°C, and the enzyme activity in the supernatant was measured. The POD activity was measured as the rate of decomposition of H_2_O_2_ by POD, with guaiacol as the hydrogen donor, by spectrophotometrically measuring the rate of color development at 436 nm due to guaiacol oxidation (Lin and Kao, 1999). The CAT activity was determined based on potassium permanganate titration (Wang, 2005). The APX activity was measured according to Nakano and Asada (1981), with slight modifications. The APX activity was recorded as the decrease in absorbance at 290 nm for 1 min. The reaction mixture for APX consisted of 50 mM potassium phosphate buffer (pH 7.0), 1 mM H_2_O_2_, 0.1 mM EDTA, 0.5 mM ascorbic acid, and the enzyme extract in a total volume of 3 ml. GR activity was assayed according to Foyer et al. (1997). The reaction mixture consisted of 50 mM phosphate buffer (pH 7.8), 0.1 mM EDTA, 0.2 mM NADPH, 1 mM GSSG, and the enzyme extract. The activity was determined as the rate of decrease in the absorbance of NADPH at 340 nm. The activity is expressed as U min^-1g-1^ FW.

#### ASA–GSH Pool

The GSH pool was determined according to Anderson et al. (1992), with slight modifications. Approximately 0.2 g of fresh leaves was homogenized in 2 ml of 5% sulfosalicylic acid and centrifuged at 20,000 *g* for 20 min at 4°C. The supernatant was then neutralized with 0.5 M K-phosphate buffer (pH 7.5). The reaction solution contained 0.6 mM 5,5-dithiobis-(2-nitrobenzoic acid) (DTNB), 0.2 mM NADPH, 50 mM KH_2_PO_4_ buffer, 2 mM EDTA (pH 8.0), 1 U of GR, and 0.1 ml of the extract. Total GSH was evaluated based on the rate of absorption change at 412 nm. GSSG was determined after the removal of GSH via 2-vinylpyridine derivatization. A specific standard curve for GSH was used. GSH was calculated by subtracting GSSG from the total GSH concentration. The contents of reduced ASA were assayed according to Kampfenkel et al. (1995). The assay of reduced ascorbate was based on the reduction of Fe3^+^ to Fe2^+^ by ASA. The Fe2^+^ complex that was formed with 2,2′-bipyridyl was measured at 525 nm in a spectrophotometer. The DHA+ASA content was determined by measuring the increase in absorbance at 525 nm caused by dithiothreitol (DTT).

#### Tiller Dynamics and Yield Components

The number of tillers was counted manually after transplanting, and similar plants in each barrel were selected and tagged for measurements of tiller dynamics. The newly initiated tillers of each plant were checked every 7 days. At maturity, panicle number was recorded from the remaining barrels, and 4 hills were selected and only one hill per barrel from every repeat was analyzed. The numbers of filled spikelets and unfilled spikelets was counted for every panicle. The filled spikelets were then oven-dried at 80°C to constant weight to determine grain weight. The grain-filling percentage was calculated as follows: 100 × filled spikelet number/total spikelet number. The grain weight per hill was calculated as follows: panicles × spikelets × grain-filling percentage × grain weight.

### Statistical Analysis

Data processing and the analyses were conducted using Microsoft Excel 2010. The variance analysis was performed using SPSS 17.0 for Windows to test for differences among treatments. The means of treatments were compared based on an LSD test at the 0.05 probability level (*P* < 0.05).

## Results

**FIGURE 1:**
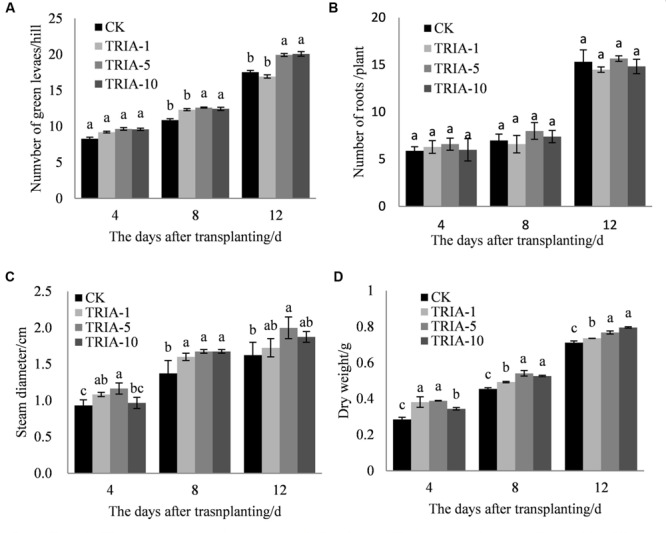
**Effects of different concentrations of TRIA on green leaf number per hill **(A)** and roots **(B)**, steam diameter **(C)**, and dry weight **(D)** after transplanting.** CK: 0 μM; TRIA-1: 1 μM; TRIA-5: 5 μM; TRIA-10: 10 μM. Segments represent the ± SEM (*n* = 3). Means followed by different letters indicate statistically significant differences (LSD, *P* < 0.05).

The influence of the application of exogenous TRIA was significant on the number of green leaves (**Figure 1B**), stem diameter (**Figure 1C**), and dry weight (**Figure 1D**) after transplanting compared to these parameters in the controls. Of the three TRIA concentrations applied, spraying with 5 and 10 μM provided better results than spraying with 1 μM for the number of green leaves and dry weight, but no differences were found in the number of green leaves or dry weight between plants that were treated with 5 or 10 μM TRIA. The stem diameters of plants that were grown with 5 μM TRIA were larger than the diameters from other treatments. However, an effect of exogenous TRIA on root regeneration after transplanting was not observed (**Figure 1A**).

**FIGURE 2:**
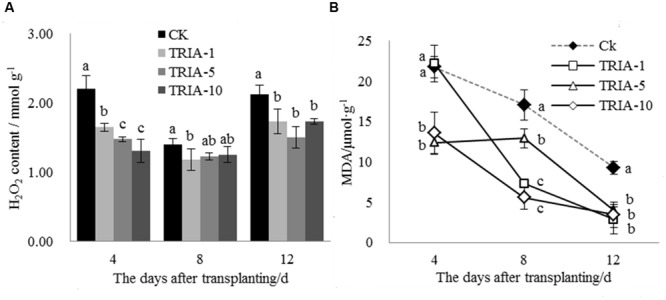
**Effects of different concentrations of TRIA on H_2_O_2_**(A)** and MDA **(B)** content after transplanting.** CK: 0 μM; TRIA-1: 1 μM; TRIA-5: 5 μM; TRIA-10: 10 μM. Segments represent the ± SEM (*n* = 3). Means followed by different letters indicate statistically significant differences (LSD, *P* < 0.05).

The H_2_O_2_ levels in the leaves after transplanting changed as a result of TRIA treatment (**Figure 2A**). The TRIA spray treatments resulted in a marked decrease in the H_2_O_2_ content at 4 days, and spraying plants with 10 μM TRIA provided the best results, decreasing the H_2_O_2_ content by 40.62% (**Figure 2A**). The treatments sprayed with TRIA had lower H_2_O_2_ contents than the control at 8 and 12 days; however, no difference in the H_2_O_2_ content was observed. Membrane lipid peroxidation with respect to the MDA content was significantly decreased by TRIA spraying compared with that of the controls (**Figure 2B**); the 1, 5, and 10 μM TRIA treatments decreased the MDA content by 69.42, 56.78, and 63.04%, respectively, at 12 days after transplanting (**Figure 2B**).

**FIGURE 3:**
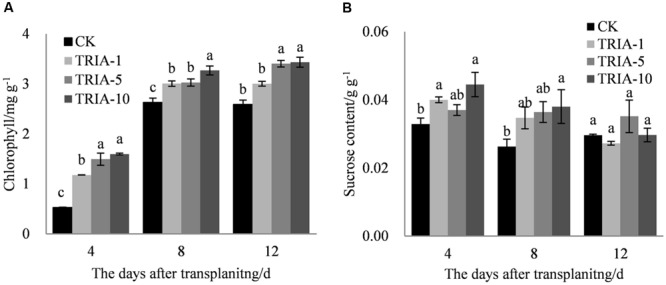
**Effects of different concentrations of TRIA on chlorophyll **(A)** and sucrose content **(B)** after transplanting.** CK: 0 μM; TRIA-1: 1 μM; TRIA-5: 5 μM; TRIA-10: 10 μM. Segments represent the ± SEM (*n* = 3). Means followed by different letters indicate statistically significant differences (LSD, *P* < 0.05).

The exogenous application of TRIA significantly improved the chlorophyll content after transplanting compared to the content of the control (**Figure 3A**). TRIA applied at 10 μM caused a significant increase in the total chlorophyll content, exceeding the control by 66.94, 19.54, and 24.62% at 4, 8, and 12 days, respectively (**Figure 3A**). No difference in the chlorophyll content was found between plants treated with 5 and 10 μM TRIA (**Figure 3A**). The application of TRIA significantly increased the sucrose content compared to that of the control, and the 10 μM treatment provided the best results, increasing sucrose content by 26.01 and 30.86% at 4 and 8 days, respectively (**Figure 3B**).

**FIGURE 4:**
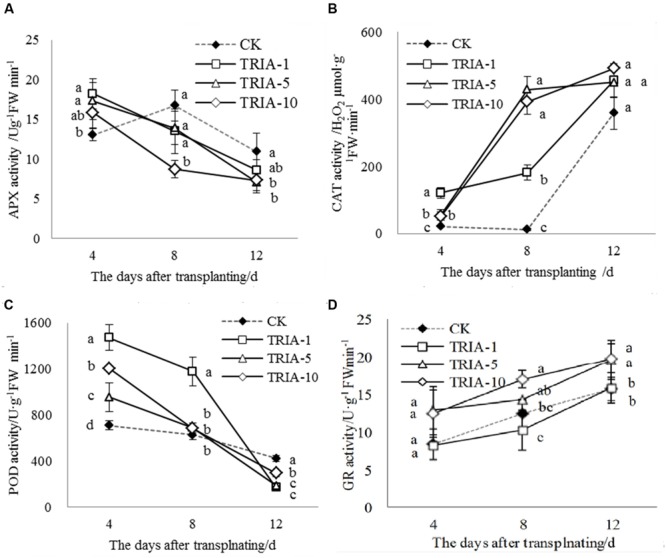
**Effects of different concentrations of TRIA on APX **(A)**, CAT **(B)**, POD **(C)** and GR **(D)** activity after transplanting.** CK: 0 μM; TRIA-1: 1 μM; TRIA-5: 5 μM; TRIA-10: 10 μM. Segments represent the ± SEM (*n* = 3). Means followed by different letters indicate statistically significant differences (LSD, *P* < 0.05).

The activities of APX, CAT, POD, and GR during the recovery stage after transplanting were affected by the application of TRIA (**Figure 4**). In plants treated with TRIA, the activity of APX decreased over time and was significantly higher than that of the control at 4 days after transplanting (**Figure 4A**). However, the activity of APX in the control plants peaked and then decreased at 8 days but was greater than that in plants treated with TRIA at 8 and 12 days after transplanting (**Figure 4A**). Across all days, the application of TRIA, compared to the effects in the control, markedly increased the activity of the antioxidant enzyme CAT, and the 10 μM concentration showed the best results, increasing the activity by 147.33, 3130.94, and 25.47% at 4, 8 and 12 days, respectively (**Figure 4B**). In addition, treatment with TRIA increased the CAT activity over time after transplanting, while the control showed a decreased and then subsequently increased activity at 8 days after transplanting (**Figure 4B**). Similarly, the activity of POD decreased over time and showed considerable improvement in the TRIA-treated plants. The POD activity of the TRIA-treated plants was markedly higher than that of the controls at 4 days, and it then rapidly decreased (**Figure 4C**). The activity of GR increased over time, and with the application TRIA at 10 μM, its activity was significantly higher than that of the control, increasing by 48.22, 36.11, and 26.41% at 4, 8, and 12 days, respectively (**Figure 4D**).

**FIGURE 5:**
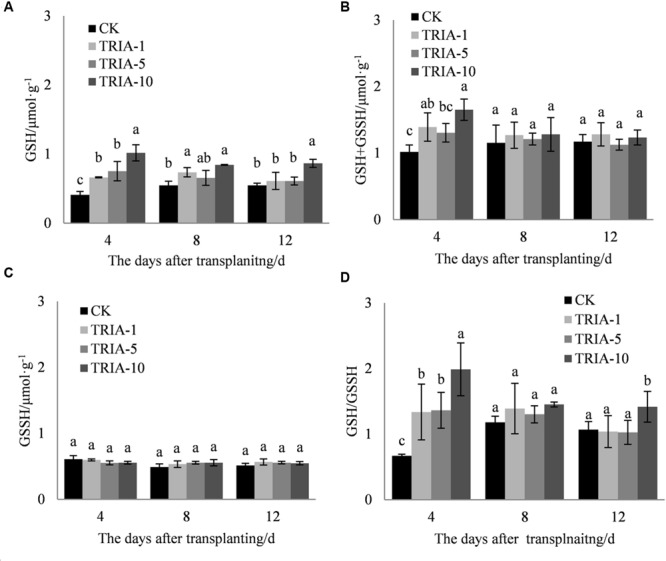
**Effects of different concentrations of TRIA on GSH **(A)**, GSSH+GSH **(B)**, and GSSH **(C)** and the GSH/GSSH ratio **(D)** after transplanting.** CK: 0 μM; TRIA-1: 1 μM; TRIA-5: 5 μM; TRIA-10: 10 μM. Segments represent the ± SEM (*n* = 3). Means followed by different letters indicate statistically significant differences (LSD, *P* < 0.05).

The application of TRIA increased the levels of total glutathione (GSH+GSSH) and GSH and the GSH/GSSH ratio compared to the values of the control (**Figures 5A,B,D**) during the recovery stage after transplanting. Importantly, the three treatments to which TRIA was applied had significantly increased GSH and GSH+GSSH levels compared to the control, and the treatments receiving the 10 μM spray had higher levels than those receiving the 1 and 5 μM treatments, exceeding the control by 148.12, 54.68, and 58.11% and 38.31, 9.82, and 5.02% at 4, 8, and 12 days, respectively. Furthermore, the application of TRIA significantly affected the GSH/GSSH ratio after transplanting; of the three TRIA treatments, the application of 10 μM, compared to the control, increased this value by 66.25, 18.83, and 24.71% at 4, 8, and 12 days, respectively.

**FIGURE 6:**
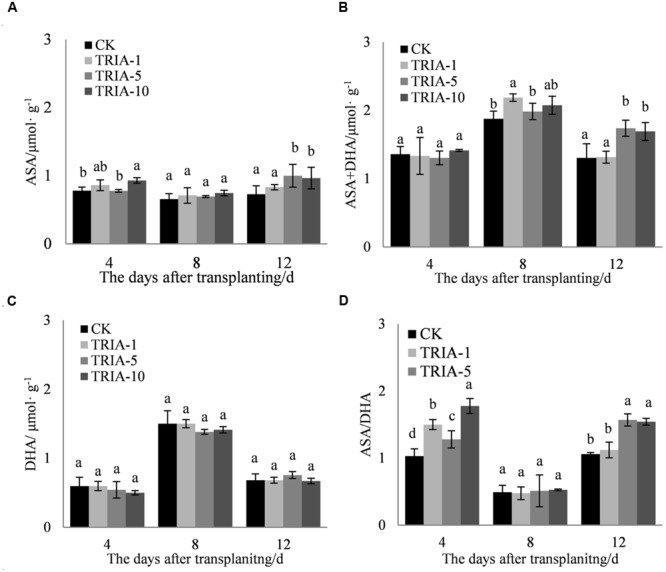
**Effects of different concentrations of TRIA on ASA **(A)**, ASA+DHA **(B)**, and DHA **(C)** and the ASA/DHA ratio **(D)** after transplanting.** CK: 0 μM; TRIA-1: 1 μM; TRIA-5: 5 μM; TRIA-10: 10 μM. Segments represent the ± SEM (*n* = 3). Means followed by different letters indicate statistically significant differences (LSD, *P* < 0.05).

The application of TRIA affected the levels of ASA and ASA+DHA and the ASA/DHA ratio during the recovery stage after transplanting (**Figures 6A,B,D**). The application of TRIA increased the level of ASA after transplanting relative to that of the control, but only the 10 μM treatment caused any significant difference, exceeding the control by 19.11, 13.72, and 32.30% at 4, 8, and 12 days, respectively (**Figure 6A**). The application of TRIA also increased the level of ASA+DHA at 8 and 12 days after transplanting. TRIA significantly increased the ASA/DHA ratio compared to that of the control, and the 10 μM treatment had a more significant effect than did the 1 and 5 μM, exceeding the control by 72.24% at 4 days after transplanting.

**FIGURE 7:**
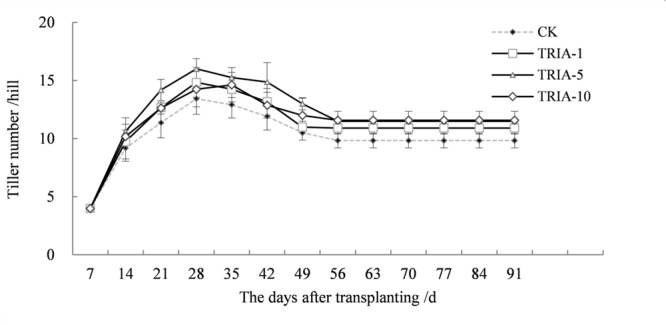
**Effects of different concentrations of TRIA on the number of tillers per hill after transplanting.** CK: 0 μM; TRIA-1: 1 μM; TRIA-5: 5 μM; TRIA-10: 10 μM. Segments represent the ± SEM (*n* = 3).

The application of TRIA influenced the number of tillers, causing an earlier tiller occurrence after transplanting and increasing the number of tillers (**Figure 7**). Of the three TRIA concentrations, 10 μM TRIA was the most effective, increasing the number of tillers by 17.80% compared to the control plants at maturity.

The application of TRIA had a significant effect on grain filling and grain yield per hill at maturity (**Table 1**). TRIA significantly increased grain filling and grain yield per hill compared to the results in the control group, and the 10 μM treatment was more effective than the 1 and 5 μM treatment, exceeding the control by 7.84 and 16.49%. However, TRIA application had no effect on the number of spikelets per panicle or the grain weight at maturity.

**Table 1:** Yield components of the different TRIA treatment concentrations.

Treatment	Panicles hill^-1^	Spikelets panicle^-1^	Grain filling(%)	Grain weight (mg)	Grain yield (g hill^-1^)
CK	9.83 a	108.28 a	86.51 a	28.00 a	27.72 b
TRIA-1	10.92 ab	108.30 a	93.29 b	28.67 a	31.59 a
TRIA-5	11.33 b	105.89 a	91.97 b	28.37 a	31.31 a
TRIA-10	11.58 b	105.59 a	91.58 b	28.53 a	31.96 a

*CK: 0 μM; TRIA-1: 1 μM; TRIA-5: 5 μM; TRIA-10: 10 μM. Means followed by different letters indicate statistically significant differences (LSD, *P* < 0.05).*

## Discussion

Currently, there are reports concerning the effects of TRIA on photosynthesis and growth (Chen et al., 2003; Naeem et al., 2009). Furthermore, the foliar application of TRIA enhances crop production under conditions of abiotic stress, including water stress (Muthuchelian et al., 1997), salt stress (Perveen et al., 2013), and acidic mists (Muthuchelian et al., 2003). In this study, foliar-applied TRIA promoted rice seedling growth during the recovery stage, as shown by the increased dry weight, green leaf number and stem diameter of the plants, and spraying with 10 μM TRIA gave the best results (**Figure 1**). Interestingly, TRIA had no effect on the number of roots, which is in contrast to findings from a previous study (Tantos et al., 2001). These differences might be due to a disrupted hormone balance caused by machine transplantation, which injures the roots (Vysotskaya et al., 2001). Furthermore, the TRIA-mediated increase in growth could also be due to the role of TRIA in modulating the activities of different enzymes (Perveen et al., 2011) and enhancing the photosynthetic rate of the plants (Singh et al., 2012). The mechanism underlying the TRIA-mediated regulation of plant growth is unclear, but some results have confirmed that TRIA plays a positive role in regulating plant growth. In this study, we observed that treating plants with TRIA improves the occurrence of low tillers and significantly increases the number of panicles at maturity (**Figure 7**). Zhao reported that low-position tillers exhibit a higher production capacity than do high-position tillers (Zhao, 1993). Our study also demonstrates that the treatment of plants with TRIA results in greater grain filling and grain yield per hill than was observed in the control plants (**Table 1**), which is in accordance with findings of Zhang et al. (2010).

Plant photosynthesis is greatly affected by transplant shock, which decreases rice seedling photosynthesis after transplanting (Yamamoto, 1989; Yamamoto and Matsumoto, 1990). The increase in photosynthesis caused by TRIA has been previously reported to be an important plant response to this treatment, which might be associated with increased leaf chlorophyll content (Naeem et al., 2009, 2010; Singh et al., 2012). In the present study, the foliar spraying of TRIA markedly increased the sucrose content compared to that of the control (**Figure 3B**), which was possible due to an improvement in photosynthesis caused by the application of TRIA. Decreased Chl content under water stress has been previously reported (Muthuchelian et al., 1997). However, TRIA application counteracted this effect and increased the total Chl content (**Figure 3**), which might be due to an increase in the synthesis of both of Chl a and Chl b (Muthuchelian et al., 1995). Furthermore, chlorophyll degradation is associated with a rapid and large accumulation of superoxide radicals and H_2_O_2_ (Djanaguiraman et al., 2009). The results of the present study show that the application of TRIA decreased the peroxidation of membranes by reducing the H_2_O_2_ and MDA content and that it improved the Chl content compared to that of the control after transplanting (**Figures 2** and **3**), indicating that TRIA is an efficient ROS scavenger and membrane stabilizer. Khan et al. reported that TRIA may play a significant role in inhibiting the lipid peroxidation of biological membranes by acting as an antioxidant compound (Khan et al., 2009). Some evidence has shown that TRIA is involved in the regulation of the antioxidant defense system (Rajasekaran and Blake, 1999; Borowski and Blamowski, 2009). The results presented here suggest that TRIA can strongly protect the structural integrity of the membranes against oxidative damage, which may be achieved by modulating the antioxidant system by directly or indirectly eliminating ROS.

Low levels of ROS production may act as a signal, while high levels result in oxidative stress. The rapid and efficient detoxification of ROS is vital to avoiding damage at the cellular level (Mittler, 2002). Therefore, plants possess a well-defined enzymatic antioxidant defense system to protect against ROS. The activity of CAT increased over time during the recovery stage after transplanting, and the application of TRIA resulted in markedly higher CAT activities than were observed in the control, but with different patterns of variation (**Figure 4B**). The activity of POD decreased over time, and the application of TRIA resulted in significantly higher POD activities than were observed in the control at 4 days but with the opposite effect at 12 days (**Figure 4C**), which was possibly due to a rapidly recovery in TRIA-treated plants after transplanting. The results show that CAT and POD have different sensitivities in response to the adverse effects of transplanting, which impairs the activity of CAT and activates POD. Thus, we speculate that POD plays an important role in scavenging H_2_O_2_ during the recovery stage after transplanting. TRIA-treated plants had significantly increased activities of CAT and POD compared to the unsprayed plants after transplanting (**Figure 4**), demonstrating that TRIA relieves the damage of abiotic stress by modulating the antioxidant enzymes that are involved in eliminating ROS. These results agree with the findings of Lu and Zhu (2006), who reported that TRIA increased the activities of peroxidase (POD) in Glycine max under water stress. More recent studies also suggest that TRIA plays an important role in antioxidant protection under conditions of water stress (Thind, 1991) and salt stress (Çavuşoğlu et al., 2008; Perveen et al., 2011). TRIA is believed to play a significant role in inhibiting the lipid peroxidation of biological membranes by acting as an antioxidant compound (Khan et al., 2009).

In addition to enzymatic antioxidants, plant cells can also be protected from ROS damage by non-enzymatic antioxidants. One of the most important antioxidant systems is the ascorbate–glutathione (ASA–GSH) metabolic cycle in plants (Foyer and Noctor, 2011). However, relatively few studies have focused on the regulation of the ASA–GSH cycle by TRIA. In this cycle, ascorbate peroxidase (APX) catalyzes the reduction of H_2_O_2_ into water, with reduced ascorbate (ASA) serving as an electron donor (Aravind and Prasad, 2005). Our results demonstrate that the application of TRIA alters the activity of APX during the recovery stage after transplanting (**Figure 4A**), suggesting that TRIA induces detoxification through ASA-metabolizing pathways. APX activity is directly dependent on ASA availability (Anjum et al., 2014). Increased levels of ASA and the ASA/DHA ratio were observed in TRIA-treated seedlings during the recovery stage (**Figure 7**) as a result of the increased GSH level (**Figure 5**). GSH is central to the regeneration of ASA in the ASA–GSH cycle. The redox state of GSH is maintained by glutathione synthetases and GR, which are involved in the biosynthetic and recycling pathways of GSH, respectively (Foyer and Noctor, 2011). In the current study, a TRIA-induced increase of GSH in machine-transplanted rice during the recovery stage after transplanting was accompanied by a significant increase in GR activity (**Figures 4** and **5**), suggesting that TRIA switches on the regeneration of the GSH pool by activating GR. TRIA also significantly increased the GSH/GSSG and ASA/DHA ratios during the recovery stage after transplanting (**Figure 5**), indicating that TRIA maintains an appropriate oxidative and reductive environment in cells by regulating the GSH/GSSH and ASA/DHA ratios (Barrameda-Medina et al., 2014). Our results demonstrated that the application of TRIA increases the GSH and ASA levels and the GSH/GSSG and ASA/DHA ratios, which could help to alleviate transplant-shock-induced oxidative damage (**Figures 2**, **5**, and **6**).

The foliar application of TRIA during the recovery stage can reduce the negative influence of transplant shock on machine-transplanted rice. A concentration of 10 μM TRIA used as a foliar spray was the most effective treatment during the recovery stage and at maturity. In conclusion, the present results clearly demonstrate that the foliar application of exogenous TRIA alleviates transplant shock, shortens the recovery stage and increases grain yield by regulating antioxidant enzymes and the redox states of ASA and GSH, thus, reducing ROS damage and maintaining photosynthetic capacity. Our future research will focus on the plant hormones, especially CK, which is the root of synthetic hormones, that are involved in the TRIA-regulated plant response to transplant shock.

## Author Contributions

GL conceived and designed the experiments. YD, SW, ZL, ST, CD, and LC provided experimental opinion and help. XL, QZ, and YL performed the experiments. XL analyzed the data and wrote the paper.

## Conflict of Interest Statement

The authors declare that the research was conducted in the absence of any commercial or financial relationships that could be construed as a potential conflict of interest.
